# Successful Surgical Intervention and Remarkable Recovery in a Child With Traumatic Spondyloptosis

**DOI:** 10.7759/cureus.59494

**Published:** 2024-05-01

**Authors:** Siddharth K Patel, Sohael Khan, Ashutosh Lohiya, Kashish Khurana, Kashyap Kanani, Harsh M Thesia

**Affiliations:** 1 Department of Orthopaedics, Jawaharlal Nehru Medical College, Datta Meghe Institute of Higher Education and Research, Wardha, IND; 2 Department of Medicine, Jawaharlal Nehru Medical College, Datta Meghe Institute of Higher Education and Research, Wardha, IND

**Keywords:** spine, lumbosacral dissociation, spinal fusion, spondyloptosis, traumatic

## Abstract

More than 100% of the traumatic subluxation of one vertebral body over another in the coronal or sagittal plane is known as traumatic spondyloptosis, which typically results in the contusion of the spinal cord. It is an uncommon yet severe spinal column injury. Here, we present traumatic lumbosacral spondyloptosis at the L5 and S1 levels with complete spinal cord compression with paraplegia and bowel and bladder involvement. The patient underwent posterior spinal fusion (delta fixation) and decompression. The patient improved his motor and sensory deficits at one-month follow-up. By the eighth-month follow-up, the patient had recovered entirely from his motor and sensory deficits and was stable for the entire year.

## Introduction

Traumatic spondyloptosis is an uncommon injury caused by high-energy trauma that leaves the injury unstable and in need of surgical stabilisation and restoration [1,2]. Moreover, severe abnormalities arising from translational spinal injuries disturb the vertebral column and surrounding components, causing deficiencies in neurological function [3]. Even though traumatic spondyloptosis is supposed to cause severe neurological impairment, there have been reports of patients with intact neurological systems [4]. Traumatic spondyloptosis is the term used to describe grade V spondylolisthesis, characterised by the complete displacement of a superior vertebral body over the inferior one, resulting in non-congruence of the two end plates.

Here we present a patient with L5-S1 spondyloptosis who underwent surgery with a single-staged posterior surgical approach in which delta fixation was done as it was difficult to reduce the L5 spondyloptosis body. Spondyloptosis, the most translating severe spine injury, causes contusion of all the posterior structural components of the vertebral column and the paravertebral soft tissues surrounding it, leading to significant biomechanical instability. Individuals who suffer from this type of injury usually suffer from severe neurological injuries with bowel and bladder involvement.

## Case presentation

A male patient, age 15, who had no prior medical history or surgical procedures, came to our emergency room complaining of severe low back pain and weakness in the bilateral lower limb with bowel and bladder involvement. He was involved in a traffic accident in which the autorickshaw overturned. The patient fell outside the autorickshaw and hit a divider on the road, taking its impact on his lower back, following which he had severe pain and was not able to move his lower limb.

Post-injury, he presented with pain over his lower back, an inability to move both his lower limbs, urinary retention, faecal retention, and a loss of motor and sensory sensation in the lower limb. He was fully conscious on admission. He had no perianal sensation and rectal tone was absent. He had muscle power of 0/5 and had hypoesthesia below the level of S1 bilaterally. According to the American Spinal Injury Association (ASIA) Impairment Scale, a typical neurological scale used to assess the sensory and motor levels impacted by spinal cord injury, the patient was assigned Grade C. 

**Figure 1:**
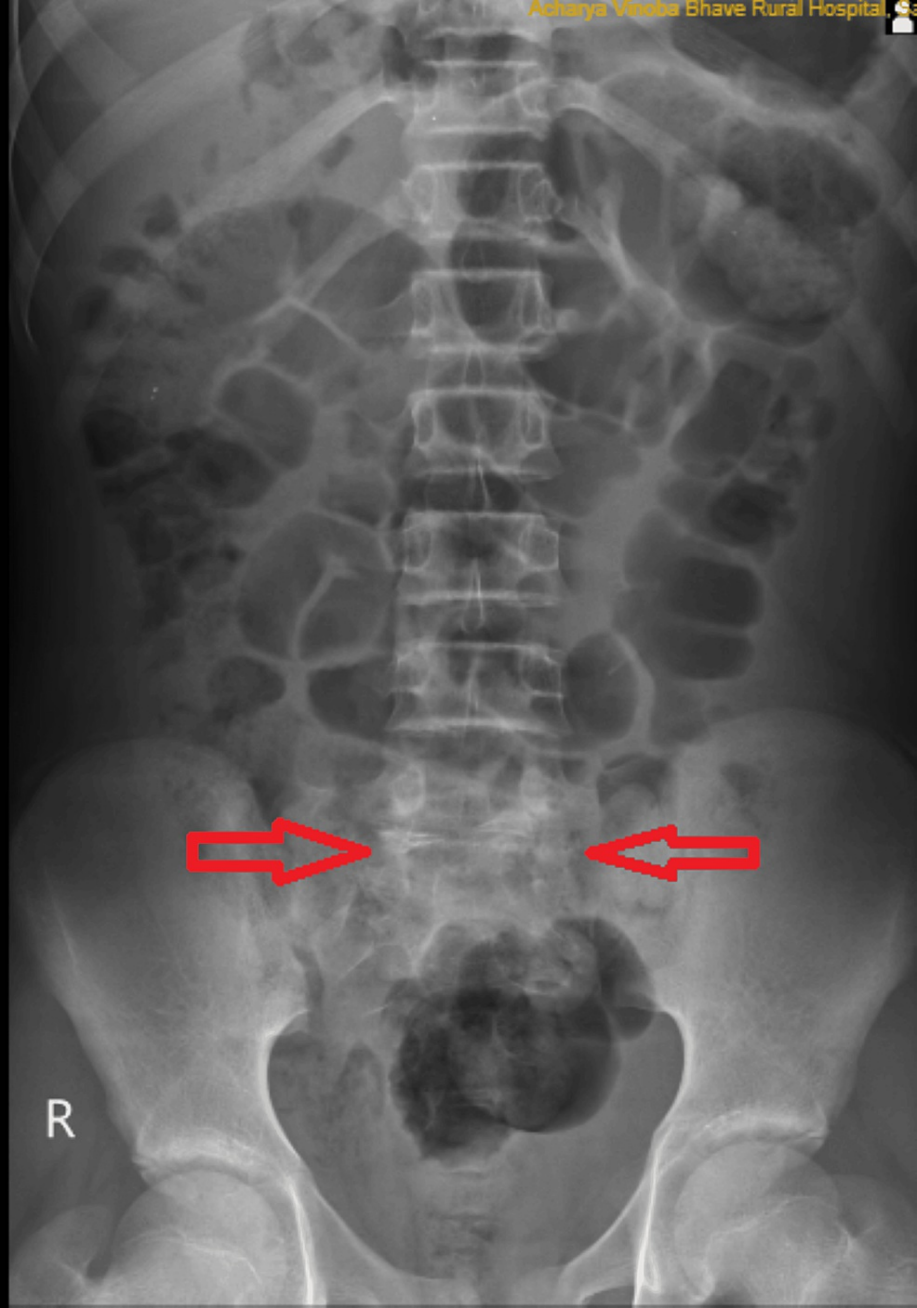
Anterior-posterior X-ray of the lumbar spine; initial injury X-ray at the time of presentation

**Figure 2:**
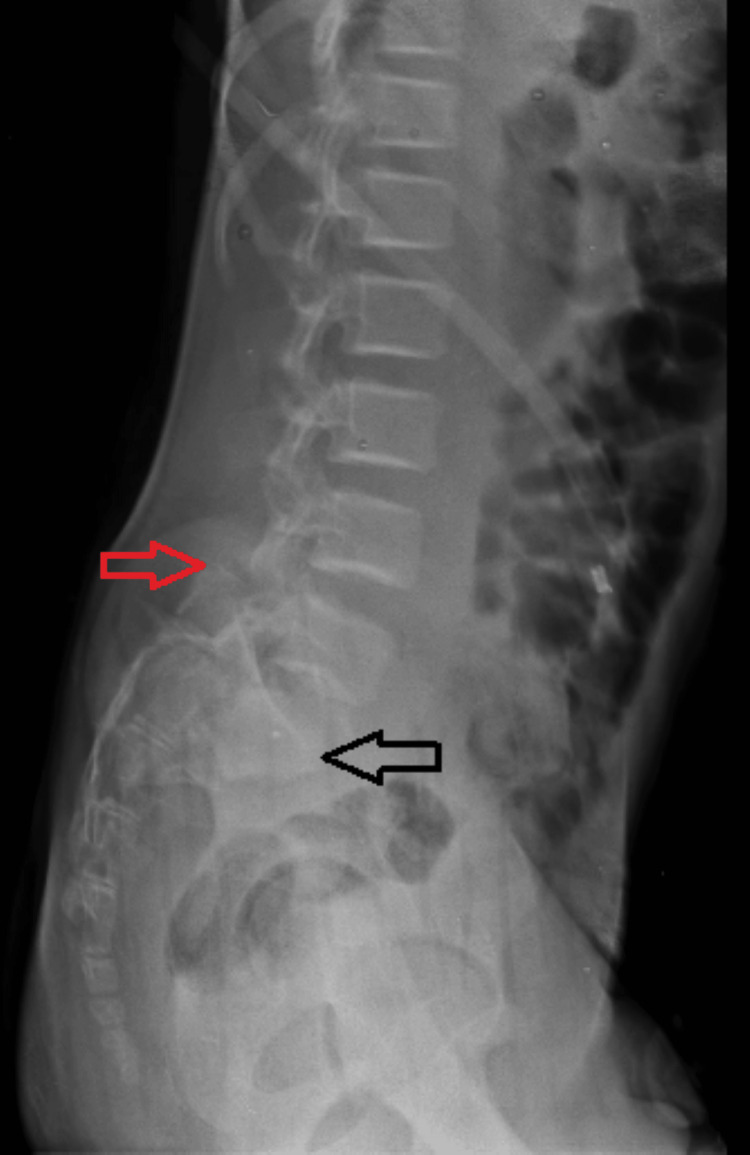
Lateral X-ray of the lumbar spine showing spondyloptosis at L5- S1 level where red arrow indicates S1 vertebra and black arrow suggests L5 vertebra

A lumbosacral spine X-ray performed on the patient was suggestive of traumatic spondyloptosis at L5-S1, which was visible in the anteroposterior and lateral views, as shown in Figures 1, 2.

**Figure 3:**
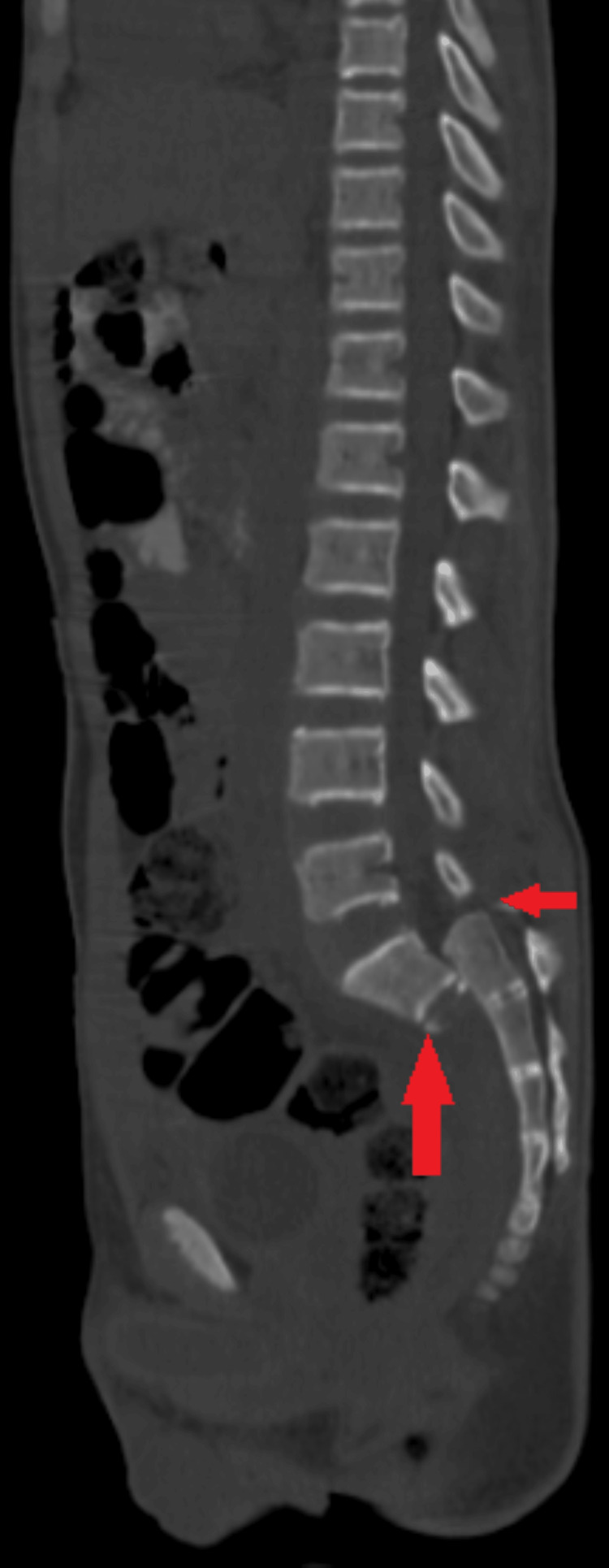
CT lumbar spine sagittal sequence demonstrating spondyloptosis at the L5-S1 level

A computed tomography scan (CT scan) of the lumbar spine determined that lumbosacral dissociation was caused by pedicle fractures of L4 and L5 and spondyloptosis at L5-S1 as shown in Figure 3.

**Figure 4:**
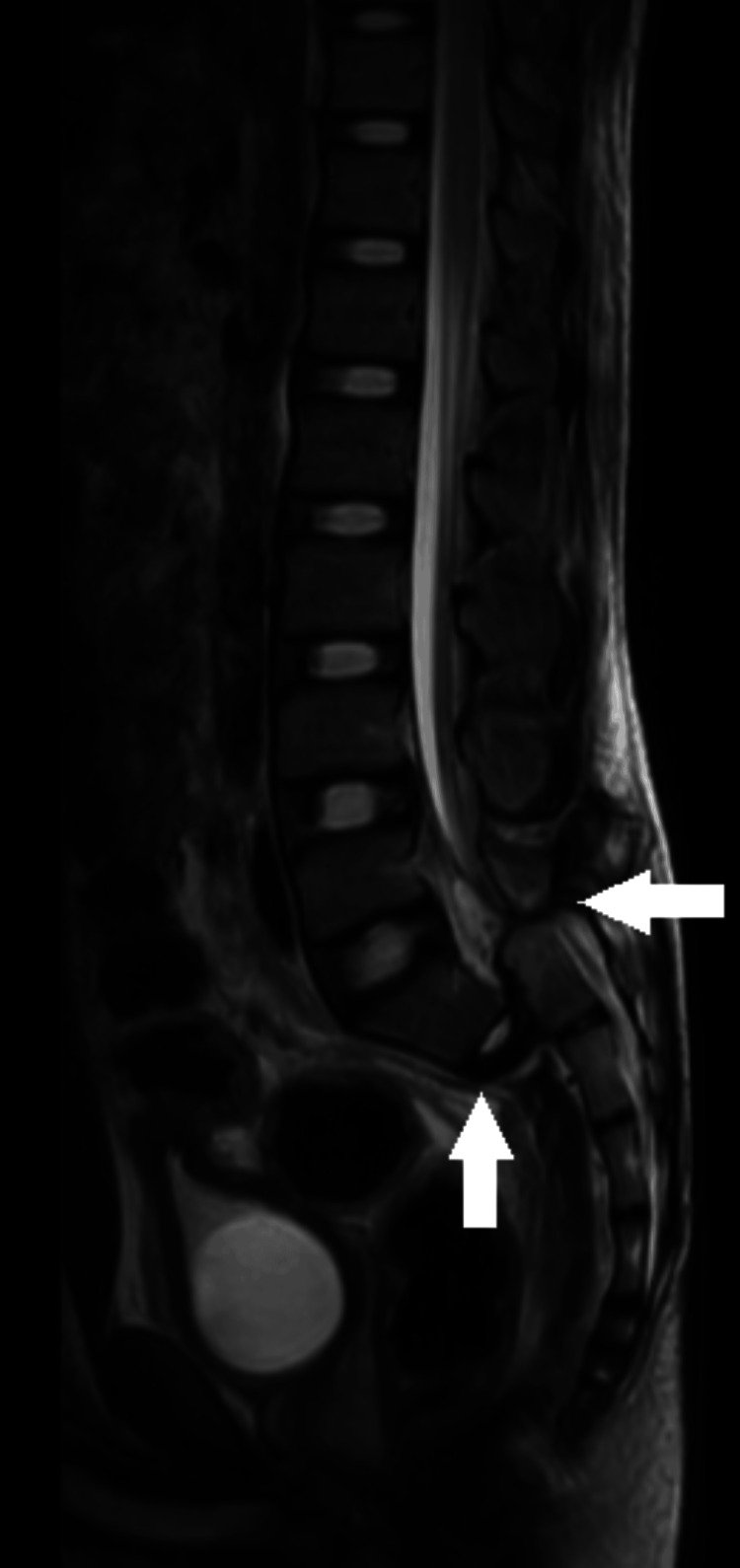
T2-weighted MRI image of the lumbar spine sagittal sequence demonstrating disruption of the anterior and posterior longitudinal ligaments with complete disruption of the L5-S1 intervertebral disc T2: transverse relaxation time

A magnetic resonance imaging (MRI) of the lumbosacral spine was performed, which revealed anterior and posterior longitudinal ligament disruption, and spondyloptosis at the L5-S1 level with transverse process fracture of L4 and L5 with complete cord compression/transection at the L5-S1 level as shown in Figure 4.

After a complete examination, fixation of the spine was planned for the patient, with due risk explained to him. The patient was positioned in a prone position on the Wilson frame. The posterior spine was accessed through a posterior approach through a longitudinal incision, exposing the L4-S1 vertebrae.

Intraoperatively, lumbar decompression was done from below L4 to the S1 level. The plan was to reduce the L5 vertebral body and restore the alignment, but unfortunately the reduction was not completely possible from the posterior approach because while reducing the body, the spinal cord was getting stretched. But the spinal cord was intact despite the velocity of the trauma. So we decided to go ahead with delta fixation for the same, in which we planned to fix S1 with the L5 level to achieve an in-situ construct. 

**Figure 5:**
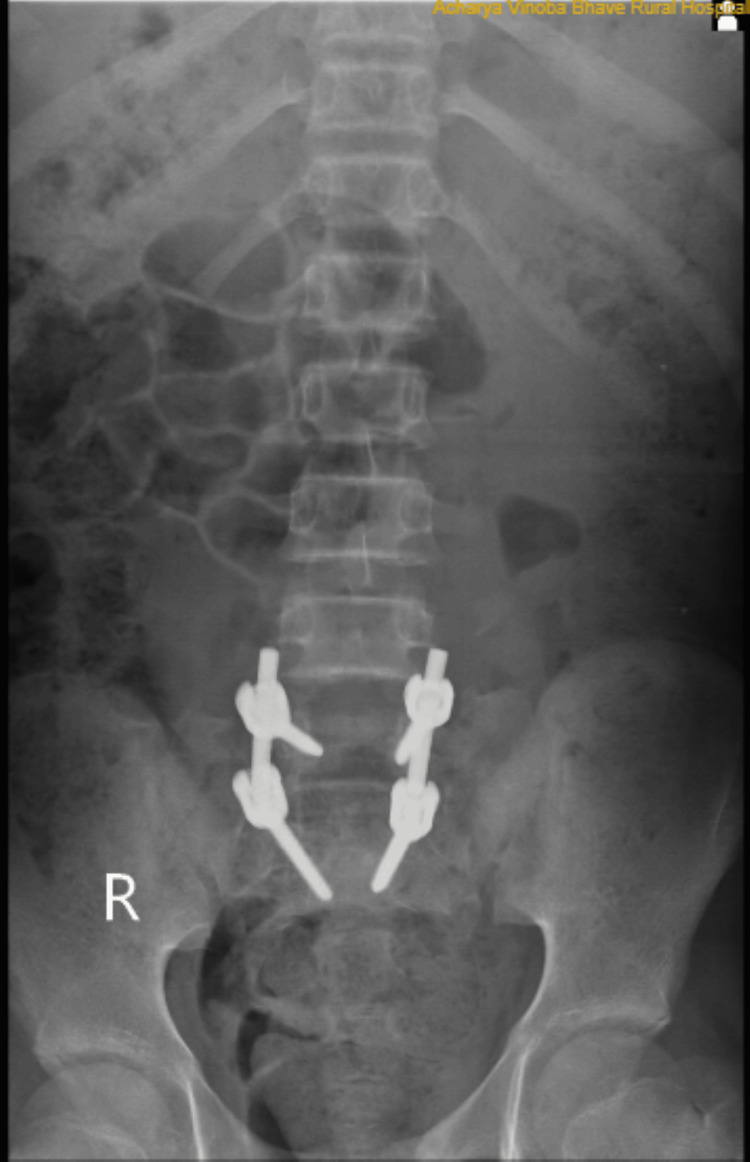
Post-operative anterior-posterior X-ray of the lumbar spine, showing spine fusion of the L4-S1 vertebrae

**Figure 6:**
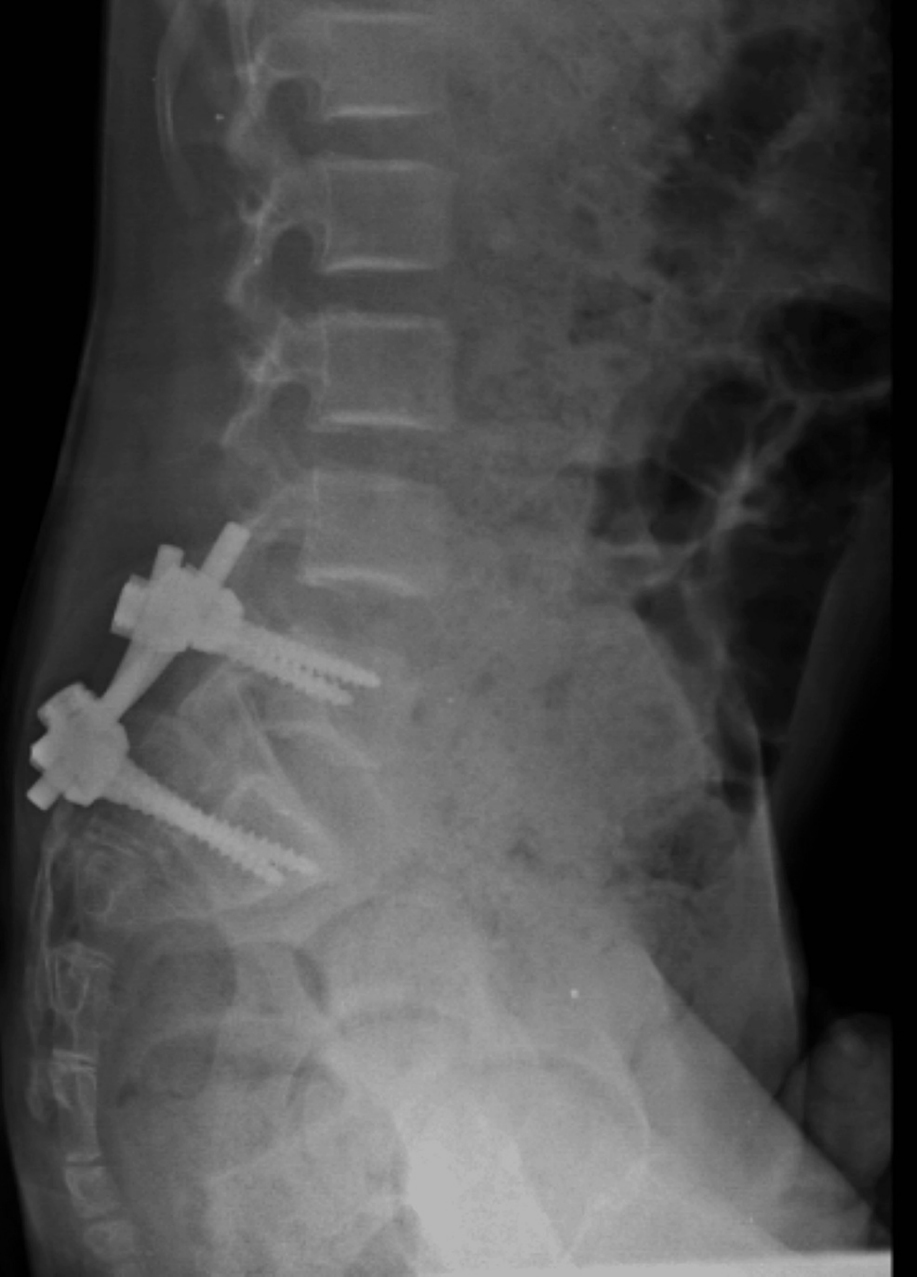
Post-operative lateral X-ray of the lumbar spine, showing spine fusion of the L4-S1 vertebra

Fixation at the L4 and S1 levels was done with a 5.5-mm polyaxial pedicle screw and connecting rods. Laminectomy was done from the L4-S1 level. Delta fixation gave the unstable spine a stable construct and spinal cord was visualised completely and there were no intraoperative complications. An anterior-posterior/lateral lumbosacral spine X-ray collected on post-operative day 2 showed the alignment of the lumbosacral spine with the implant in situ as shown in Figures 5, 6.

The patient was started on dexamethasone 8 mg intravenous injection on a tapering dose. After 24 hours of the surgery, the motor power began to be restored with his hip flexion, extension, abduction, and adduction on the left and right sides, improving to muscular power grade 3/5. Power became 5/5 gradually in 15 days. In addition, he continued to use a silicone catheter and experienced bladder and bowel issues. Two months after the surgery, the patient began to regain his bladder and bowel function. The patient was advised complete bed rest for six weeks. The patient was mobilised with the help of a walker and was planned for rehabilitation and general exercises. Eight months post-operation, the patient visited us in our outpatient department, walking with complete control of the bowel and bladder. 

**Figure 7:**
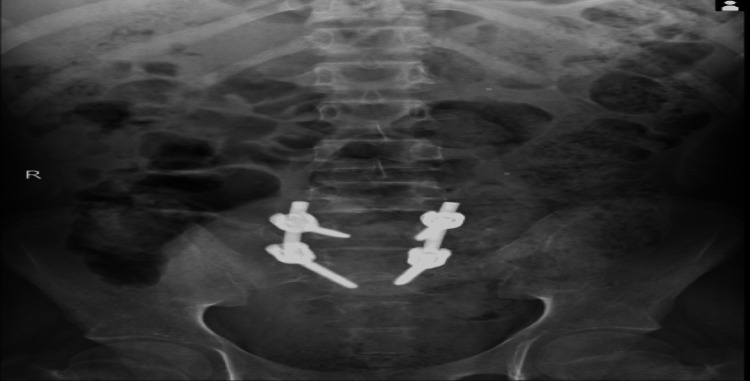
Anterior-posterior X-ray of the lumbar spine at the eighth-month follow-up demonstrating evidence of fusion

**Figure 8:**
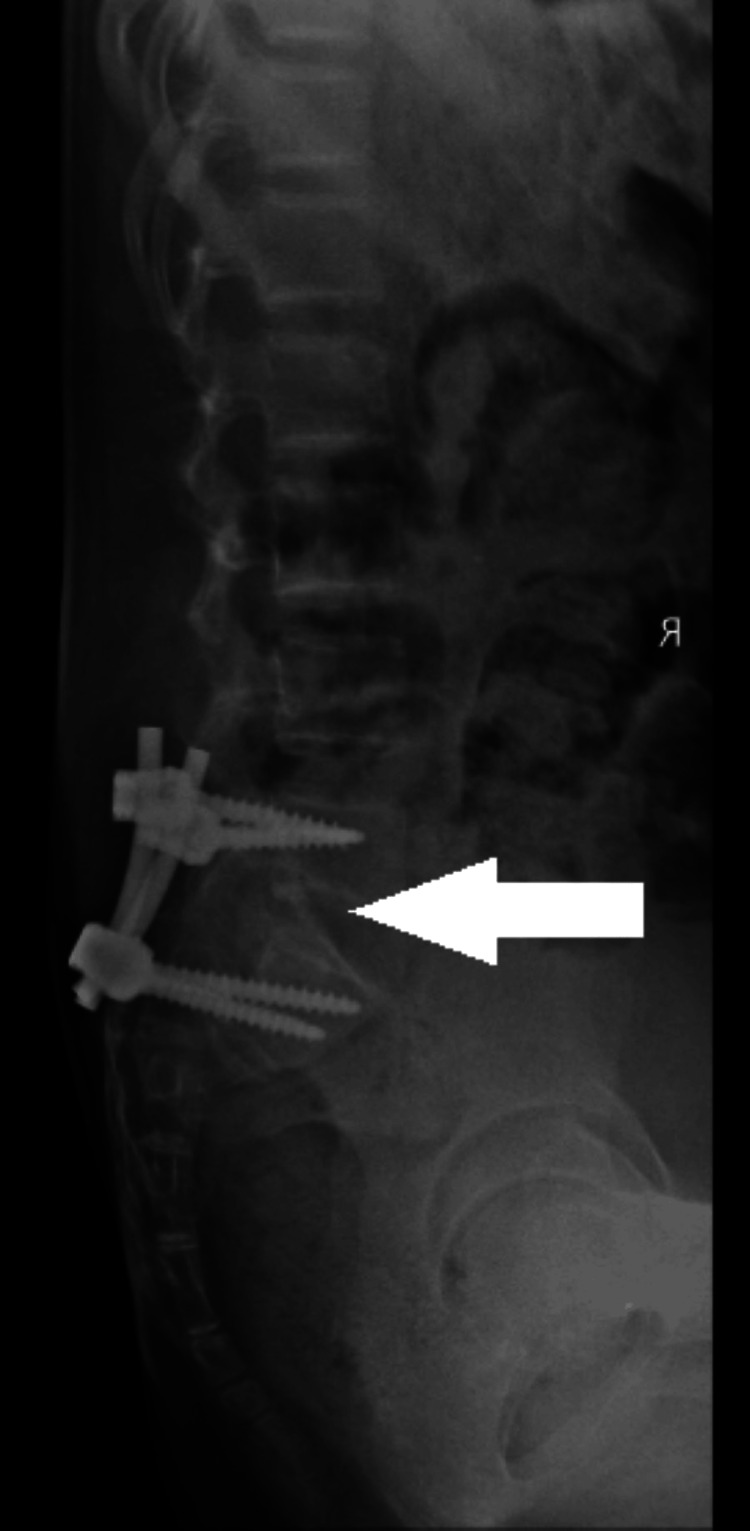
Lateral X-ray of the lumbar spine at the eighth-month follow-up demonstrating evidence of fusion (white arrow)

We repeated the anterior-posterior/lateral lumbar-sacral spine X-rays at the eighth-month follow-up, which showed satisfactory lumbosacral spine alignment and evidence of fusion as shown in Figures 7, 8.

## Discussion

The incidence of lumbar spondyloptosis is just 5% according to literature and it is the rarest and most severe kind of spondylolisthesis [5]. Surgery is necessary for treating traumatic spondyloptosis to decompress the spinal canal, reduce the spine abnormalities, and restore stability. Because of the intricate nature of the injury and its accompanying problems, therapy usually entails a multi-phase process [6]. However, this instance was unique because a single-stage operation was performed and fixation was done for spondyloptosis and to stabilise the spine. In our case, we did a spinal fusion of L4-S1 for the stabilisation of the spine in the posterior approach, which showed an effective improvement in restoring stabilisation and neurologic outcome.

A break or disruption in one or more of the vertebral bones that comprise the spinal column is referred to as a spine fracture. Numerous factors, including trauma from falls or accidents, as well as underlying medical problems that weaken the bones, can cause these fractures. A frequent surgical technique for treating unstable spine fractures or abnormalities is posterior instrumentation. To stabilise the broken or damaged vertebrae, metal rods, screws, or other hardware are positioned along the posterior portion of the spine. This equipment system supports and facilitates the appropriate healing of the fracture by acting as an internal brace. The goals of posterior instrumentation are to realign the spine, release compressed neural structures, and encourage fusion, or the mending of the damaged vertebrae. This surgical strategy is frequently used in conjunction with other methods, including procedures such as bone grafting or decompression treatments, to provide the best possible results and reduce the discomfort caused by spinal fractures.

The posterior approach to the spine has fewer related difficulties and comorbidities as compared to the anterior approach [7]. A typical surgical technique for treating a variety of spinal disorders, such as deformities, degenerative illnesses, and some fractures, is posterior-only fusion. Using this method, the spine is accessed from the posterior and equipment (such as screws and rods) is inserted to stabilise the damaged vertebrae. Long-term stability and support are provided by the solid bony bridge that is eventually created between the vertebral segments as a result of the fusion process, which promotes the formation of new bone tissue. Compared to conventional open operations, posterior-only fusion has the advantage of a less intrusive technique, which may shorten patient recovery periods, surgical times, and blood loss. But it is crucial to thoroughly assess every case, taking into account elements like the particular spinal disease, the severity of the deformity, and the patient's general health and bone quality [8,9]. Spondyloptosis, which is a severe form of spondylolisthesis, is a condition in which one vertebra slides forward over the lower vertebra. A complete displacement or dislocation of one vertebral body over another, resulting in a notable spinal deformity and possible neurological consequences, is precisely referred to as spondyloptosis.

Although surgery has been shown to be beneficial in treating spondyloptosis, there is a chance that it could cause iatrogenic damage to the spinal cord and strain the cauda equina, which could lead to neurologic impairment [10]. It has been proposed that a rod and screw system for posterior instrumentation can be an effective way to reduce, stabilise, and decompress L5-S1 spondyloptosis [11]. Another surgical option is a two-stage procedure in which the first stage is spinal fusion by anterior approach, and the second stage is lumbar decompression and facetectomy [12,13].

Spondyloptosis should be minimised as soon as feasible to improve the likelihood of neurological recovery [11]. Neurologic recuperation varies greatly and is primarily influenced by the extent of the damage. Within 24 hours following surgery, a neurological improvement was seen in this instance. The best results are obtained with surgical reconstruction since it helps with rehabilitation and restores the stability of the spinal column. It is crucial to remember that the degree of anatomical damage discovered during surgery determines the prognosis for lumbar spondyloptosis more than the radiologic evaluation of the injury [14]. The posterior technique utilised in our patient's one-stage short-segment decompression, reduction, fixation, and fusion has been shown to provide favourable outcomes.

## Conclusions

Traumatic spondyloptosis is a rare type of injury. Mostly, patients with these types of injury present with neurological deficit. The posterior single-stage approach to this type of injury gives immediate decompression to the spinal cord and direct visualisation of the spinal cord as compared to anterior plus posterior approach. Fixation of this type of injury can be decided intraoperatively - whether to reduce the spondyloptosis or fix in situ using delta fixation technique, which we did intraoperatively, leading to a single- stage surgery. Hence, according to our experience, single-stage decompression and fixation surgery gives an immediate outcome in an acute traumatic type of spondyloptosis. 
